# An evaluation of pipelines for DNA variant detection can guide a reanalysis protocol to increase the diagnostic ratio of genetic diseases

**DOI:** 10.1038/s41525-021-00278-6

**Published:** 2022-01-27

**Authors:** Raquel Romero, Lorena de la Fuente, Marta Del Pozo-Valero, Rosa Riveiro-Álvarez, María José Trujillo-Tiebas, Inmaculada Martín-Mérida, Almudena Ávila-Fernández, Ionut-Florin Iancu, Irene Perea-Romero, Gonzalo Núñez-Moreno, Alejandra Damián, Cristina Rodilla, Berta Almoguera, Marta Cortón, Carmen Ayuso, Pablo Mínguez

**Affiliations:** 1 Department of Genetics, Health Research Institute-Fundación Jiménez Díaz University Hospital, Universidad Autónoma de Madrid (IIS-FJD, UAM), Madrid, Spain; 2 Center for Biomedical Network Research on Rare Diseases (CIBERER), Instituto de Salud Carlos III, Madrid, Spain; 3 Bioinformatics Unit, Health Research Institute-Fundación Jiménez Díaz University Hospital, Universidad Autónoma de Madrid (IIS-FJD, UAM), Madrid, Spain

**Keywords:** Genetics research, Genome informatics

## Abstract

Clinical exome (CE) sequencing has become a first-tier diagnostic test for hereditary diseases; however, its diagnostic rate is around 30–50%. In this study, we aimed to increase the diagnostic yield of CE using a custom reanalysis algorithm. Sequencing data were available for three cohorts using two commercial protocols applied as part of the diagnostic process. Using these cohorts, we compared the performance of general and clinically relevant variant calling and the efficacy of an in-house bioinformatic protocol (FJD-pipeline) in detecting causal variants as compared to commercial protocols. On the whole, the FJD-pipeline detected 99.74% of the causal variants identified by the commercial protocol in previously solved cases. In the unsolved cases, FJD-pipeline detects more INDELs and non-exonic variants, and is able to increase the diagnostic yield in 2.5% and 3.2% in the re-analysis of 78 cancer and 62 cardiovascular cases. These results were considered to design a reanalysis, filtering and prioritization algorithm that was tested by reassessing 68 inconclusive cases of monoallelic autosomal recessive retinal dystrophies increasing the diagnosis by 4.4%. In conclusion, a guided NGS reanalysis of unsolved cases increases the diagnostic yield in genetic disorders, making it a useful diagnostic tool in medical genetics.

## Introduction

The clinical and genetic heterogeneity of many genetic disorders can hinder the determination of their molecular causes, complicating diagnosis for affected families^1^. In recent years, next generation sequencing (NGS) technologies have simplified the diagnostic process. NGS has increasingly become a standard diagnostic tool in clinical practice, allowing screening for pathogenic variations in hundreds to all genes and other DNA regions^2,3^. NGS strategies include whole genome sequencing (WGS), whole exome sequencing (WES), in which only coding regions are sequenced, and sequencing of custom panels of different genes. A very demanded instance for routine genetic diagnosis is clinical exome (CE) sequencing^4–6^, which consists of sequencing large panels covering a few thousand disease-associated genes, often referred to as the Mendeliome^4–6^.

CE sequencing has been reported to be a cost-effective first-tier molecular test^6–8^. Despite these advances, the diagnostic yield of rare hereditary diseases using CE remains around 30–50% ^5^^,^^9^^,^^10^. There are several causes underlying this relatively low diagnostic yield, such as limitations of analytical methods (variant calling and annotation);^11,12^ the genetic and phenotypic diversity of some genetic disorders;^13^ knowledge gaps in gene-disease and variant-disease associations; and a lack of structured databases of these associations^14^. On the other hand, the need for manual examination by an expert molecular geneticist introduces further bias and makes analyses difficult to reproduce^15,16^. A number of studies suggest that periodic reevaluation of inconclusive cases using improved bioinformatics tools and updated variant–disease databases and completing the analysis with complementary methods such as copy-number variant (CNV) detection, can result in the discovery of new candidate mutations^2,14,17^. Others propose to explore non-coding variants using WGS^18–20^ as they can affect transcription^21^, although we still need tools to annotate regulatory regions to improve the prediction of their pathogenicity^22^. However, reanalyses are often performed at the request of clinicians or patients due to the usual overload of cases under analysts’ hands. As a result, new bioinformatics developments and new disease related knowledge are needed in order to obtain a conclusive diagnosis for the majority of the unsolved cases. In this sense, automatic reanalysis might be a helpful and cost-effective tool to complement the process of diagnosis.

In this study, we introduce a custom reanalysis pipeline (FJD-pipeline) built with state-of-the-art bioinformatics software and with updated and complete annotations from different databases. We compared the ability of this pipeline to call variants by variant type and detect causal variants against two commercial software solutions used in the primary analysis of 4953 cases with presumed inherited disease (4211 heterogeneous cases of genetic disorders, 614 inherited cancer cases, and 128 cardiovascular diseases). In addition, we performed a retrospective reanalysis of unsolved cases of three subcohorts from the CE cohort of the medical genetics service of Fundación Jiménez Díaz University Hospital (FJD-UH in Madrid, Spain).

## Results

### A framework to evaluate the performance of a custom bioinformatics pipeline as compared to DNA sequencing tests in a large cohort of patients

The medical genetics service at FJD-UH (Madrid, Spain) handles a large cohort of patients with diverse genetic diseases. Of them, 4,953 underwent targeted sequencing using several panels according to their availability and/or the suspected disease. Two of those panels were generic: TruSightOne (TSO) and Clinical Exome Solution (CES). Two other targeted sequencing panels: TruSight Cancer (TSCa), and Hereditary Cancer Solution by Sophia Genetics (HCS) were applied to hereditary cancers, and the Nextera Rapid Capture (NRC) panel was used for cardiopathies. Variant detection was performed with different commercial bioinformatics software programs depending on the sequencing panel: (1) the Illumina-pipeline (applied to TSO, TSCa, and NRC) and (2) the Sophia-pipeline (Sophia Genetics pipeline applied to CES and HCS). We developed a custom bioinformatics pipeline (FJD-pipeline) for the analysis of DNA sequencing (DNASeq) (see Materials and Methods). In order to design a filtering and prioritization algorithm that could be applied in systematic reanalysis of unsolved cases of our cohort, we first compared the overall performance of the FJD-pipeline against the commercial programs used.

**Fig. 1:**
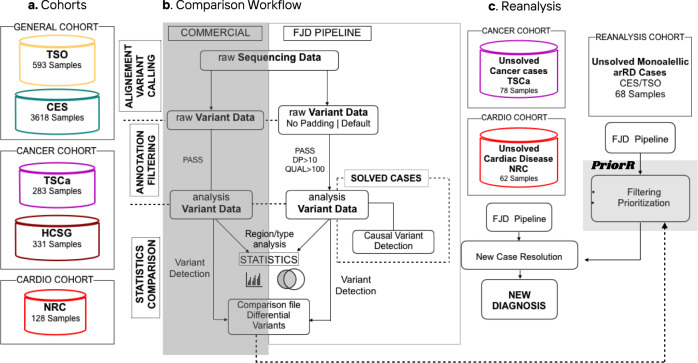
General framework of this study. **a** The different subcohorts used: the heterogeneous cohort of genetic diseases (TSO and CES), the hereditary cancer cohort (TSCa and HCS), and the cardiovascular disease cohort (NRC). **b** Workflow followed to compare the performance of the commercial pipeline and the FJD-pipeline. **c** Steps followed in the systematic reanalysis of negative (unsolved) cases.

The general workflow of the comparison between the FJD-pipeline and the commercial solutions is summarized in Fig. 1. First, we applied the FJD-pipeline to three subcohorts classified by disease composition (a heterogeneous cohort of genetic diseases, a cancer and a cardiogenetic cohorts) (Fig. 1a). Then we collected detected variants and compared to previous results the number of: (1) all detected variants, (2) detected variants classified by type (SNVs or INDELs) and selected by clinical relevance, and (3) detected variants classified by genomic region (Fig. 1b). Since the FJD-pipeline was able to provide variants in extended regions (padding) of the targeted regions, comparison with the commercial software programs was performed using both, that is, all variants provided by the FJD-pipeline and, for an objective comparison, the variants from the targeted regions only (no-padding). There is no other difference between our two protocols but the application to extended regions. Next, we evaluated the capacity of the FJD-pipeline to detect the causal variants reported for the solved cases using the commercial solutions. Simultaneously, unsolved cases from the TSCa and NCR cohorts sequenced between May and July of 2018 were reanalyzed using the FJD-pipeline (Fig. 1c). Based on the former comparisons and the reanalysis results, the differential variant detection capabilities of the FJD-pipeline were implemented in a reanalysis protocol as DNA variant filtering and prioritization tasks. Thus, we performed a new reassessment using the same sequenced data of selected cases from the general cohort based on a custom prioritization method (Fig. 1c). The following sections report the comparisons of the FJD-pipeline and commercial software programs for every subcohort, the evaluation using causal variants and, finally, the results of reanalysis.

### Comparison of the performance of the FJD and commercial pipelines in a heterogeneous cohort of patients

Samples from 4211 individuals included in a heterogeneous cohort of patients were sequenced using CE as part of their genetic diagnosis. Out of the 4211 samples, 593 were sequenced using TSO and analyzed by means of the Illumina-pipeline, achieving a conclusive molecular diagnosis in 213 cases (35.9%). The other 3618 samples were analyzed using CES and analyzed by means of the Sophia-pipeline. Of these, 1265 were solved, reaching a diagnostic rate of 34.9%. Retrospectively, we analyzed all the 4211 cases with the FJD-pipeline in order to compare its performance with their respective commercial pipelines, as well as to evaluate its ability to detect causal variants.

**Fig. 2:**
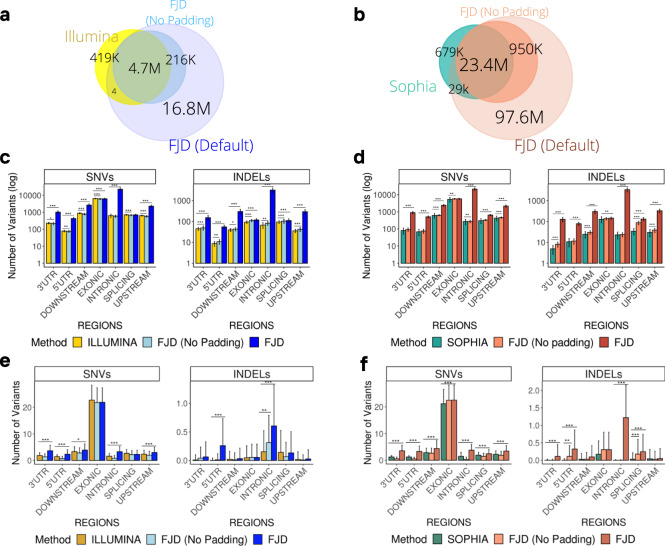
Comparison of the performance of the FJD-pipeline and the commercial pipelines in the detection of variants in samples from the general cohort. Venn diagrams showing the overlap of variants detected the commercial pipelines, (**a**) Illumina and (**b**) Sophia, and the FJD-pipeline (with and without padding applied). Bar plots represent the mean of the number of SNVs and INDELs detected in samples by (**c**) the Illumina-pipeline and the FJD-pipeline, and (**d**) the Sophia-pipeline and the FJD-pipeline, in different genomic regions. Bar plots show the average number of clinically relevant variants detected by (**e**) the Illumina-pipeline and (**f**) the Sophia-pipeline and the FJD-pipeline, in each type of genomic region. Clinically relevant variants are selected as those annotated by the ClinVar database as “*pathogenic*”*,* “*likely pathogenic*”*,* “*uncertain significance*” or a combination of just those categories, VUS are filtered by allele frequency (GnomAdg_AF_POPMAX < 0.1). The distributions are shown using the mean and standard deviation for visual ease. A *t* test was applied for the comparisons. Significant differences between values are indicated by asterisks: *p < 0.05, **p < 0.01, ***p < 0.001.

Figure 2a shows the overall comparison between FJD and Illumina pipelines, both sharing 88% of the detected variants, FJD identifying less variants but surpassing Illumina when the extended regions are included (Supplementary Table 1). Global comparison of the number of variants detected distinguishing SNVs and INDELs are described in Supplementary Fig. 1A, briefly FJD-pipeline retrieves fewer SNVs and more INDELs (FC = 0.94 and 1.14, *p* values = 3.26e–10 and 8.43e–08) considering the same regions and overload both types using our padding approach (FC = 3.73 and 10.97, *p* values = 2.50e–63 and 1.82e–57). (Supplementary Tables 2 and 3). The differences were especially pronounced in intronic and untranslated regions (UTRs), for SNVs, and also in downstream and upstream regions for INDELs (Fig. 2c) (Supplementary Tables 4 and 5). Considering only clinically relevant variants, we observe a similar behavior of the FJD-pipeline, retrieving slightly fewer exonic SNVs (FC = 0.96, n.s) and more non-coding SNVs when extended regions are applied (Fig. 2e and Supplementary Fig. 1B) (Supplementary Tables 6–9).

The same trend is observed in the comparison between Sophia and FJD pipelines, having roughly 23 million of common detected variants (92.8% of the total, Supplementary Table 1) and with a considerable increase when extended regions are added by the FJD-pipeline (Fig. 2b). In addition, there were ~29,000 variants found by both the commercial and the FJD-pipeline but not by FJD in targeted regions due to a partial match between INDELs in which the breakpoint lay outside the bed file (see Methods). In terms of SNVs and INDELs, the FJD pipeline in targeted regions reported slightly fewer SNVs but more INDELs (FCs = 0.98 and 1.21, *p* values = 1.71e–03 and 2.12e-19) on average (Supplementary Fig. 1C and Tables 2 and 10). If SNVs and INDELs are detached in genomic regions, the FJD-pipeline detects more variants in non-exonic regions, especially in intronic and UTRs for SNVs, and in downstream and upstream regions for INDELs (see FC and *p* values in Supplementary Tables 11 and 12). Again, focusing on clinically relevant variants, FJD-pipeline gets a few more SNVs differences that increase with extended regions considered, especially in non-coding regions (Fig. 2f and Supplementary Fig. 1D) (Supplementary Tables 13–16). Remarkably, restricted to the same targeted regions, FJD-pipeline reports more pathogenic, likely pathogenic or VUS INDELs in intronic regions (Fig. 2f).

### Comparison of pipeline performance in the hereditary cancer and cardiogenetics datasets

A total of 614 hereditary cancer samples were sequenced at FJD-UH as part of the patients’ diagnostic process. Two different cancer-related gene panels were used to construct the library: 283 samples with the Illumina TSCa panel and 331 samples with Sophia Genetics HCS Panel solution. In total, there were 77 (27.2%) and 64 (19.33%) solved cases with the Illumina and Sophia pipelines, respectively. Additionally, a custom panel of cardiogenetics designed using NRC was used to sequence 128 patients with cardiovascular disease, as well prescribed as part of their diagnosis. The sequence data was processed with the Illumina-pipeline. A total of 36 patients out of 128 were diagnosed with a causal variant (28.13%). Samples from these cohorts were analyzed retrospectively with the FJD-pipeline.

**Fig. 3:**
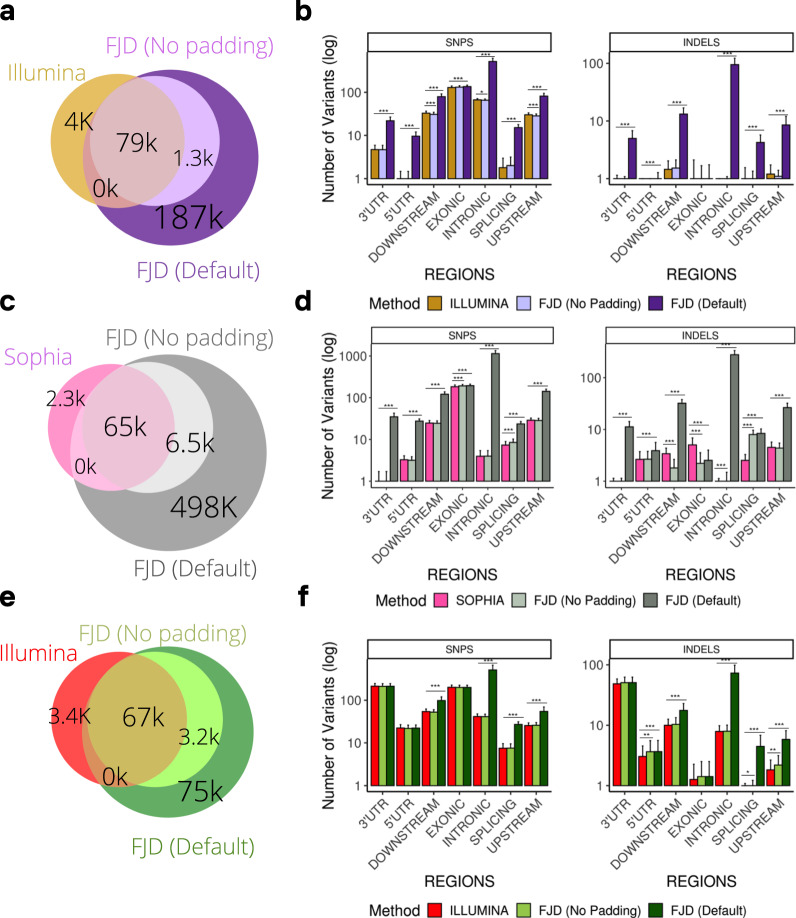
Comparison of variants detected between the FJD-Pipeline and the commercial pipelines of Illumina and Sophia in the cancer and cardio cohorts. Venn diagrams showing the overlap of variants detected between the FJD-pipeline and the commercial pipeline: (**a**) Illumina -TSCancer, (**c**) Sophia panel, (**e**) Illumina-Nextera panel. The bar plots show the average number of variants (variant count) detected by the FJD-pipeline and the commercial pipelines (**b**) Illumina-TSCancer Panel, (**d**) Sophia, (**f**) Illumina- Nextera Panel, in each type of genomic region, the distributions are shown using the mean and standard deviation for visual ease. Significant differences between values are indicated by asterisks: *p < 0.05, **p < 0.01, ***p < 0.001.

In respect to these three subcohorts, the performance of the FJD-pipeline in comparison with commercial pipelines was analogous to what had already been reported for the general cohort. Whereas the Illumina-pipeline (TSCa and NRC) detected slightly more number of variants than the FJD-pipeline when no padding was applied, the Sophia-pipeline (HSC) detected slightly fewer variants in the same conditions (Fig. 3a, c, e, Supplementary Table 1). However, including extended regions, the FJD-pipeline detected a larger number of variants in all three subsets (TSCa, HCS and NRC) (Fig. 3a, c, e, Supplementary Table 1). If numbers of SNVs and INDELs per sample are considered, the FJD and both commercial pipelines behaved similarly when the FJD-pipeline is restricted to the targeted regions, while if the padding is applied, the FJD-pipeline increased the detection of SNVs and INDELs (Supplementary Tables 17–20, Fig. 2). Finally, when SNVs and INDELs were broken down by genomic regions, an increase in the detection of non-exonic variants was observed in the FJD-pipeline compared to both commercial pipelines for the three subsets of data (Fig. 3b, d, f) (Supplementary Tables 21–26).

### FJD-pipeline performance in detecting causal variants reported by the commercial software in the solved cases

Focusing on differences in detecting the causal variant in the heterogeneous cohort, from the 213 solved cases analyzed using TSO, the FJD-pipeline detected all 270 causal variants (100% efficiency). In the cases sequenced using CES, 1,265 individuals obtained a conclusive diagnosis, with a total of 1506 causal variants detected in the initial analysis using the Sophia-pipeline. Of them, 1501 variants were detected by the FJD-pipeline (99.66%), and five were not reported (Table 1), three of them being the same variation in *PAX6* which is preceded by a homopolymer repeat where the sequencers tend to make the post-homopolymer error^23^. In addition, a variant in the gene *SPRY4* was rejected by the FJD-pipeline on the grounds that it did not meet the diploidy criteria, having 18% of variant allele fraction (VAF), while Sophia-pipeline mapping obtained VAFs within the inclusion criteria. Finally, one variant in the gene *ABCC6* was filtered out by our quality filters due to low mapping quality.

**Table 1:** Discordant variants only detected by the Sophia pipeline in the CES cohort.

Sample	Gene	Transcript	Nucleotide	Type	Explanation
17-0531	*PAX6*	NM_000280.5	c.1268 A > T	Non-stop	Low AD/Homopolymer
19-1853	*PAX6*	NM_000280.5	c.1268 A > T	Non-stop	Low AD/Homopolymer
20-1426	*PAX6*	NM_000280.5	c.1268 A > T	Non-stop	Low AD/Homopolymer
13-2707	*SPRY4*	NM_030964.4	c.23-2 A > C	Essential splice site	Low AD
19-1532	*ABCC6*	NM_001171.6	c.474 + 5 G > C	Extended splice donor site	Low MQ

Regarding the cancer and the cardiogenetics cohort, the FJD-Pipeline found the 77 causal variants identified by the panel TS Cancer and studied with Illumina-pipeline, together with the 64 causal variants identified by the HCS panel and studied with the Sophia-pipeline. The FJD pipeline also detected the entire collection of previously reported causal variants in the cardiogenetics cohort (36 in total). Thus, the FJD-pipeline showed 100% efficiency detecting the causal variants of these cohorts.

Taking into account the results from the five subcohorts described, the FJD-pipeline showed an efficiency of 99.74% detecting the causative variant in previously solved cases.

### Reanalysis of unsolved cases of cancer and cardiogenetics subcohorts

In an initial evaluation of the capacity of the full FJD-pipeline to provide an updated diagnosis in a retrospective reanalysis of unsolved cases, we analyzed all unsolved cases of those sequenced between May and July of 2018 of two subcohorts of the three described: the hereditary cancer cohort (78 samples) and the cardiogenetics cohort (62 samples). From this reanalysis, the molecular geneticists detected candidate variants not previously reported by the Illumina-pipeline; these were later confirmed as causal variants solving two dominant cases in the cardiogenetics cohort and two other dominant cases in the hereditary cancer cohort. Variants found in cases of the cancer cohort were in genes: *RB1* and *NF1*, both coherent with the phenotype of the patients: Retinoblastoma and Neurofibromatosis type 1, respectively. Additionally, variants found in individuals of the cardiogenetics cohort were in genes: *MYBPC3* and *KCNH2*, both consistent with the pathologies investigated: Arrhythmia and Hypertrophic cardiomyopathy. All four reported variants were splicing variants that lay outside the targeted regions (see Table 2 for details). Thus, the increase in the diagnostic yield of the FJD-pipeline on reanalysis was 2.5% and 3.2% for cancer and cardiovascular disease, respectively.

**Table 2:** Causative variants only detected by the FJD-pipeline in the cancer and cardiovascular disease datasets as part of a systematic reanalysis of negative cases.

Panel	Sample	Gene	Transcript	Nucleotide	Type	Inheritance	zygosity	Phenoty	Region	ACMG	ACMG Criteria	GnomAD AF
TSCa	18-0744	*RB1*	NM_000321.2	c.1049 + 3 A > G	SNV	AD	HET	Retinoblastoma	Splicing	Likely Pathogenic	PM2, PP3, PP5	-
	18-0871	*NF1*	NM_001042492.2	c.7190-2 A > C	SNV	AD	HET	Neurofibromatosis type 1	Splicing	Pathogenic	PVS1, PM2,PP3	-
NRC	18-0910	*MYBPC3*	NM_000256.3	c.1928-2 A > G	SNV	AD	HET	Arrhythmmia Disorder	Splicing	Pathogenic	PVS1, PP5, PM2,PP3	-
	18-2249	*KCNH2*	NM_000238.4	c.1557 + 1 G > C	SNV	AD	HET	Hypertrophic cariomyopathy	Splicing	Pathogenic	PVS1, PP5, PM2, PP3	-

### Reassessment of cases using a prioritization algorithm

**Fig. 4:**
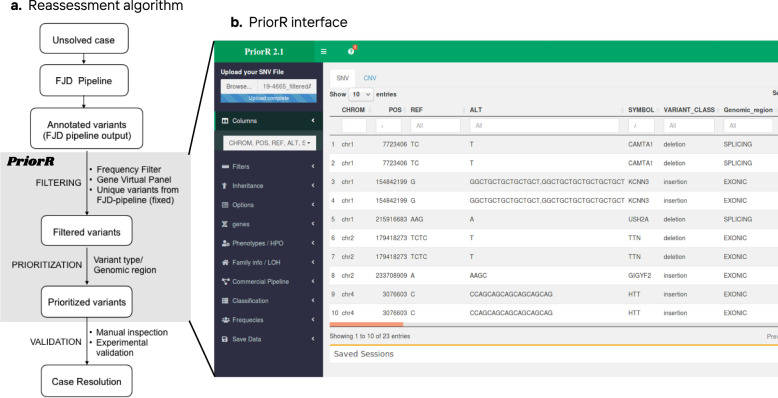
Reanalysis algorithm in PriorR. **a** Describes the algorithm followed in the reassessment of the selected negative cases from the general cohort. The output of the FJD-pipeline is read and analyzed in PrioR where variant filtering and prioritization is carried out; this process is followed by a validation in case of candidate variants. **b** PriorR interfaces for SNV analysis.

We developed a filtering and prioritization algorithm (Fig. 4a) based on the outcome of the differential detection of total and clinically relevant variants and the reanalysis of unsolved cases in the cancer and cardiogenetics subcohorts, both described in previous sections. Although we reanalyzed the entire collection of unsolved cases using the full FJD-pipeline, 68 unsolved cases with autosomal recessive Retinal Dystrophy (arRD) with one allele confirmed in the first diagnosis screening were selected for reassessment by the molecular geneticists as: (1) being those patients the closest to obtain a diagnosis, and (2) having, in principle, a higher probability that the missing variant is in the sequenced region and pass the panel filter.

This analysis was carried out with PriorR (see “Methods”), a tool designed to analyze the output of the FJD-pipeline (Fig. 4b). Thus, in our protocol, the ~4000 resulting rare variants in canonical transcripts (already filtered by Maximum AF < 0.05) were subsequently filtered out by (i) AF < 0.01 and POPMAX AF < 0.01, reducing the number of variants to ~1000; (ii) variants present in the commercial vcf were filtered out, which reduced the variant number to ~300; and (iii) an in-house virtual panel of 241 inherited genes causing retinal dystrophy (IRD), remaining ~45 variants. The molecular geneticists were asked to leave the last filter fixed, whereas they were free to change the rest during reanalysis. After filtering, variants were prioritized according to the conclusions of the comparative study of the pipelines, mainly based on their type and genomic region, thus INDELs and those variants belonging to non-exonic regions were prioritized over the rest. A molecular geneticist took 12 min on average to review a single case.

The 68 selected cases of arRD were reviewed by a single geneticist; of these, 4 cases were tagged with a candidate variant, there were two cases of Leber congenital amaurosis (LCA), one case of Joubert Syndrome (JS) and one case of Retinitis Pigmentosa (RP). The variants were found in 4 known RD causing genes consistent with the reported diseases: *GUCY2D* (LCA), *CEP290* (LCA), *AHI1* (JS) and *TULP1* (RP). (Table 3), three of which had already a variant in the same gene (Supplementary Table 27). The second variant found in *GUCY2D* was confirmed as a causal variant by checking the mapping data, where both variants were in trans (Supplementary Fig. 3A), the second variant in *AHI1* was confirmed by familiar segregation (Supplementary Fig. 3B), whereas the variant in *CEP290* was confirmed as causative just based on its pathogenicity and coherence with the phenotype. Variant in *TULP1* was classified as VUS following ACMG criteria^24^ (Table 3). In summary, the diagnostic gain of the reassessment was 4.4% for this subcohort

**Table 3:** Variants found during the reanalysis of 68 cases of arRD. Three of the 4 variants were classified as pathogenic and confirmed as causal variants, 1 of the variants was however classified as VUS waiting for experimental confirmation.

Sample	Gene	Transcript	Nucleotide	Protein	Type	Inheritance	Zygosity	Phenotype	Region	ACMG	ACMG Criteria	GnomAD AF
18-0126	*GUCY2D*	NM_000180.3	c.389del	p.Pro130LeufsTer36	Del	AR	HET	Leber´s congenital Amaurosis	Exonic	Pathogenic	PVS1, PP5, PM2, PM3	0.0000174
16-0951	*CEP290*	NM_025114.4	c.1666del	p.Ile556PhefsTer17	Del	AR	HET	Leber´s congenital Amaurosis	Exonic	Pathogenic	PVS1,PS3, PP5, PP3	-
21-0476	*AHI1*	NM_017651.4	c.910dup	p.Thr304AsnfsTer6	Dup	AR	HET	Joubert Syndorme	Exonic	Likely Pathogenic	PVS1, PM2, PM3, PP5, PP3	-
07-0707	*TULP1*	NM_003322.6	c.371_394del	p.Asp124Glu131del	Del	AR/AD	HET	Retinitis Pigmentosa	Exonic	VUS	PM4, PP3, PP5, BS1, BS2	0.00198

## Discussion

Targeted genomic sequencing using broad gene panel solutions has become an essential diagnostic tool in clinical settings with heterogeneous cohorts of patients with genetic disorders^5^. Popular examples are the so-called clinical exomes, which screen a large proportion of the known genes associated with genetic diseases. Our own experience using two types of these resources (TSO and CES) shows an overall diagnostic rate of 34.7%, similar to other reported trials^5,9,10^. The main aim is to increase the diagnostic rate, as this is especially important in rare diseases where the overall rate of diagnosis, using any approach, is reported to be around 50%^25^. The usual procedure to manage unsolved cases after a first genetic examination is to propose further genomic tests to explore causal variants in very specific genomic regions, either regulatory regions (intronic, promoters, enhancers or 5ʹ/3ʹUTR) of known disease-associated genes via more specific gene panels^26,27^ or exploring wider regions (only coding or everything) via WES or WGS. However, previous studies have argued that the remaining unsolved cases do not necessarily require further sequencing to be diagnosed; conversely, the causative mutation might be on the data already produced^28^. In this regard, reanalysis of the sequenced data has become more important, improving processing and annotation of the data^1,14,29^.

Where time is the only consideration, regular updates of the variant annotation detected by the same bioinformatic software, the gain will be restricted to the new knowledge produced for every disease depending on periodic reanalyses. Several reanalysis pipelines have been published so far based on an economic analysis^1^, relaxation of variant filters, exploration of CNV alleles, and sequencing of additional family members^2^, and different reanalyses have been proposed depending on the case^29^. Herein, we propose a complementary approach consisting of detecting the gaps in the bioinformatics protocol for DNASeq analysis and introducing a secondary variant call using different software and a revised strategy, which could add new knowledge immediately.

Here, we present the FJD-pipeline, a long-term project for the systematic reanalysis of remaining unsolved cases with genetic diseases that have a primary diagnosis. Although the usual methodology to determine the accuracy of a DNASeq bioinformatics pipeline is to calculate detection metrics using reference standard sequencing results such as those provided by the Genome In a Bottle initiative^30^; in our case, the goal was to assess the ability of the FJD-pipeline to complement a first analysis performed by means of other software programs. We thus chose to compare the performance of the FJD-pipeline and the results from the commercial software used in the genetics service of the FJD-UH over our own collection of heterogeneous sequencing tests as a more informative approach.

The main outcome of the comparison between commercial and FJD pipelines was that we were able to detect similar to slightly fewer SNVs and more INDELs within targeted regions. This is valid considering all and only clinically relevant variants detected. In addition, the full FJD-pipeline application (i.e., with extended regions) can provide more causal variants within those regions, which reinforces the idea that our pipeline offers a complementary and finer analysis. In particular, the 4 causal variants found in the cancer and cardiogenetics datasets were SNVs in intronic regions flanking exons, meaning that expanding the target regions by applying padding is a simple and efficient strategy to detect the missing causative mutation. Those likely pathogenic variants found in the reanalysis cohort (arRD mono-allelic cases) are exonic INDELs, a fact that indicates that calling of INDELs by the FJD-pipeline is more efficient. The reported VUS is also an exonic INDEL. These results were implemented in a reanalysis algorithm and provided to the geneticists by means of our prioritization software, PriorR, designed to read the pipeline output, filter it, annotate it, and aid in prioritizing variants.

The main limitation of our work is that the number of unsolved cases due to the large cohort did not allow us to reassess all the tests due to limited geneticist availability and time restrictions. In addition, we are aware of the fact that the estimation of false negatives becomes challenging when performing diagnosis using target sequencing panels. In this scenario, the causative variant or variants might be in genes not yet associated with the pathology, not targeted at all or in non-coding regions where only WGS can have direct access to. This aspect was considered in the selection of cases for the reassessment as arRD monoallelic cases have, in principle, more probability to have a second allele in the targeted region. On the top of this, and probably as a consequence of, they are also the closest to receiving a conclusive diagnosis. Still, the FJD-pipeline was able to solve 7 cases where the missing causative variant was not present in the results of the analysis performed during the first diagnostic protocol. In total, we reassessed 208 cases from three different subcohorts with a combined diagnostic yield of 3.4%. On the other hand, the FJD-pipeline was not able to detect 5 causal variants, representing 0.26% of the 1,956 checked in 1658 cases.

In real practice, reanalysis of DNASeq tests in clinical settings remains challenging due to the labor by expert clinical researchers required^14^. Thus, by offering a protocol with which to prioritize only those variants not found in the commercial pipeline, we decreased the analysis time considerably. Analysis of a clinical exome might entail several hours of expert labor, thus effort is consequently put into analysing new clinical exomes rather than reevaluating old ones^14^. Hence, automation in the process of reanalysis should be a priority if reanalysis is to be routinely implemented. In light of the results of this work, we can implement a systematic and periodic reanalysis of unsolved cases, which complements the good performance of the primary analysis and facilitates analyst diagnosis. It is worth clarifying that reanalysis will under no circumstances substitute the work and expertise of clinicians, but will rather help them reduce the number of candidate variants to check using flexible and disease-specific filters and prioritization methods. We would also like to apply the knowledge created here to expand the reanalysis to consider CNV alleles that can be already calculated by a CNV detection protocol attached to the FJD-pipeline. However, CNV detection was not included in this study, as the Illumina pipeline did not have this analysis available.

Summing up, NGS technologies have improved the diagnosis of genetic disorders, though there is still a discouraging number of patients who must wait for years to be diagnosed. In this study, we confirm that reanalysis of sequencing data from unsolved cases using the latest bioinformatic algorithms and updated databases can increase the diagnostic rate of genetic diseases in clinical genetics services. A prioritization method based on the selection of variants not found by the commercial algorithms, and which highlight those variants with characteristics lessened in the commercial results, can ease and speed up the analysis by molecular geneticists, who frequently are burdened with a heavy workload. Future work will be focused on automating reanalysis, so that it will become another diagnosis tool for genetic diseases.

## Methods

### Cohort description and sample sequencing

To test the general performance of a custom bioinformatics pipeline, we included patients from three independent cohorts enrolled at the medical genetics service of FJD-UH (Madrid, Spain). First, a general cohort of 4,211 patients with distinct genetic disorders was analyzed from May 2017 to July 2020 by means of CE sequencing. Two different commercial CE approaches were used: 593 cases were analyzed using *TruSightOne (TSO*, Illumina), and 3618 cases using *Clinical Exome Solution (CES) v.2 (*Sophia Genetics), capturing coding exons and flanking regions of 4813 and 4493 disease-related genes, respectively. Annotations of the causal variants of the 213 cases and 1265 cases solved by TSO and CES, respectively, were used to test the accuracy of the custom pipeline to detect those variants.

A second cohort of 614 patients with presumed hereditary cancer (Cancer cohort), whose DNA was analyzed from January 2018 to August 2020, were also included in the study. Two different commercial targeted gene panel approaches were used: the Illumina TruSight Cancer Sequencing (TSCa) panel (283 samples) and the Hereditary Cancer Solution (HCS) kit (Sophia Genetics) for 331 samples, targeting coding exons and flanking regions of 97 and 127 cancer-related genes, respectively. Sequencing data for this cohort were used to analyze the ability of bioinformatic pipelines to call genomic variants with differential features. The sequencing data of 141 solved cases was used to study the ability of the custom pipeline to find the causal variant.

Lastly, a third cohort comprising 128 patients with presumed inherited cardiovascular disease that were analyzed from January 2018 to July 2019 was also included in the study (Cardiogenetics cohort). Samples were sequenced using a custom panel of 95 cardiovascular disease related genes designed using the Nextera Rapid Capture (NRC) library preparation approach. Data of the entire cohort were used to evaluate how efficiently the bioinformatic pipelines call genomic variants with differential features. Furthermore, sequencing data of the solved cases (*N* = 36) were used to confirm the capability of the FJD-pipeline to identify the causative mutation.

### Ethics approval

All patients signed an informed consent before participating. The project was reviewed and approved by the Research Ethics Committee of HU-FJD (Ref. 2016/ 59) and fulfils the principles of the Declaration of Helsinki and subsequent reviews.

### DNA sequencing and commercial bioinformatic analysis

All libraries from the different capture approaches described above were sequenced in the NextSeq500 system (Illumina) and were first analyzed with a commercial bioinformatics pipeline. Bioinformatic analysis from Illumina based libraries (TSO, TSCa, and NRC) was carried out by BaseSpace software (Illumina); sequence alignment to the GRCh37/hg19 assembly of the human genome was performed by the Burrows–Wheeler Aligner (BWA)^31^ and variant calling with Genome Analysis Toolkit (GATK)^32^. Annotation and variant interpretation were conducted with VariantStudio v3.0 (Illumina). This pipeline is referred to as the Illumina-pipeline.

Bioinformatic analysis, annotation, and variant interpretation of CES and HCS was carried out using the Sophia DDM platform (Sophia Genetics). Alignment was performed to the GRCh37/hg19 assembly of the human genome. This pipeline is referred to as the Sophia-pipeline.

### FJD bioinformatics pipeline

Raw sequencing reads were aligned to the GRCh37/hg19 assembly using the BWA v 0.7.15^31^ with default parameters. GATK v 4.1.2.0^32^ was used to perform insertion/deletion realignment and base quality score recalibration (BQSR). Single nucleotide variants (SNVs) and short insertion/ deletion (INDELs) were identified using the GATK HaplotypeCaller. Hard filtering was performed with two possible filters for SNVs and INDELs, respectively: *SNP_filter*: QD (Quality of Depth) <2.0, MQ (Mapping Quality) <40.0, MQRankSum < −12.5, and ReadPosRankSum < −8.0, and *INDEL_filter*: QD < 2.0, and ReadPosRankSum < −20.0. Finally, the resulting variants were annotated using the Ensemble Variant Effect Predictor (VEP, release 98)^33^ including annotations from different databases: Genome Aggregation Database (gnomAD v2.0.1)^34^, the Combined Annotation Dependent Depletion (CADD)^35^ database or Online genetic Inheritance in Man (OMIM);^36^ pathogenicity predictors: SIFT^37^ and Polyphen;^38^ splicing predictor: MaxEntScan^39^ and filtered by the filter from the Hard Filtering (FILTER = PASS), depth of coverage (DP > 10) and quality (QUAL > 100). Transcripts were defined using the RefSeq database^40^.

Bed files with specific extended regions for each sequencing protocol were used to run the pipeline so that only sequencing data overlapping those regions were considered. Each region was extended by 1000 base pairs before the start and after the end of it. The extension of the target region is referred to as padding. Although positions outside the targeted regions usually have worse coverage, all variants reported in those regions fulfilled the quality filters (QUAL > 100 and DP > 10).

The resulting variant calling format (vcf) files from both commercial pipelines were compared to the vcf files resulting from our bioinformatic analysis (FJD-pipeline). FJD-pipeline is also referred to as the full FJD-pipeline to distinguish it from the FJD-pipeline when no padding is applied. The FJD-pipeline is availableat https://github.com/TBLabFJD/VariantCallingFJD.

### Pipeline performance evaluation

*Region statistics:* Raw vcf files from the commercial pipelines were annotated with VEP and filtered using the FILTER = PASS tag from the original analysis. Variant genomic regions were extracted from VEP output. All variants in canonical transcripts were considered; if the same variant appeared in more than one canonical transcript of different genes, it was considered more than one time. *Variant counts:* In order to compare variant detection between the primary analysis performed by commercial software packages (Illumina or Sophia) and the FJD-pipeline we used vcftools^41^ to compare vcfs by pairs and extract common and different variants. *Clinically relevant variants:* Variants annotated as “*pathogenic*”*,* “*likely pathogenic*”*,* “*uncertain significance*” or a combination of just those categories by the ClinVar database were considered for calculations, VUS were also filtered by allele frequency (GnomAdg_AF_POPMAX < 0.1) *Causative variants:* Causal variants from solved cases were extracted from an internal database with diagnostic records. Causal variants for each case were searched in the annotated vcf output for both the commercial and FJD-pipeline. Overlapping INDELs were considered the same variant.

### Reanalysis of unsolved cases from the cancer and cardiogenetics subcohorts

A subset of the unsolved cases (selected by a molecular geneticist) of the cancer (78/614) and cardiovascular disease (62/128) cohorts was reanalyzed during the patients’ diagnostic process. The molecular geneticists were provided with the results from the analysis of unsolved cases by the FJD pipeline. The geneticists manually reviewed each case with the results of the FJD-pipeline. If a causative variant is found by the FJD-pipeline, we check that it is not present in the results of the primary diagnosis.

### Reassessment of cases from a subset of the general cohort

A subset of 68 patients from the general cohort with autosomal recessive retinal dystrophy (arRD) in whom a monoallelic causal variant had been detected was selected for reassessment by means of a systematic reanalysis.

The vcf files from the FJD-pipeline of the unsolved cases selected for reanalysis were converted to a text tabulated document and made available to the clinical researchers. The molecular geneticists performed the analyses using PriorR (https://github.com/TBLabFJD/PriorR/), an ad hoc prioritization software program designed to manage the output from the FJD-pipeline including filtering by diverse parameters and prioritization of variants. PriorR is implemented using Shiny R library. Molecular geneticists only reviewed variants exclusively identified by the FJD-pipeline and not by the commercial tools, filtered using PriorR. The protocol also called for application of predetermined filters to resulting variants in canonical transcripts (already filtered by Maximum AF < 0.05): (i) AF (ExAC^42^, 1000G^43^, and GnomAD^39^) <0.01 and POPMAX AF < 0.01, (ii) variants present in the commercial vcf, and (iii) an in-house virtual panel of 241 inherited retinal dystrophies (IRD) genes. The pathogenicity of candidate variants was assessed using different databases such as Varsome^44^, HGMD^45^ or Clinvar^46^.

### Statistical Analysis

A two-sided unpaired Fisher exact test was used to test the differences in the number of variants per vcf file retrieved by the different pipelines by type and genomic region. One hundred samples of each set were randomly selected to perform the test. *P* values < 0.05 were considered significant. The average fold change was calculated between the number of variants per vcf file detected by the FJD-pipeline and that detected by the different commercial pipelines.

### Reporting summary

Further information on research design is available in the Nature Research Reporting Summary linked to this article.

## Supplementary information

Reporting Summary

Supplementary Information

## Data Availability

Sequencing data have been deposited in EGA with accession number EGAD00001007022.

## Code availability

The code of the FJD-Pipeline is available at https://github.com/TBLabFJD/VariantCallingFJD and the code of PriorR at https://github.com/TBLabFJD/PriorR/.
