# Supplementary material for: An evaluation of pipelines for DNA variant detection can guide a reanalysis protocol to increase the diagnostic ratio of genetic diseases
Source: NPJ Genom Med. 2022 Jan 27;7:7. doi: 10.1038/s41525-021-00278-6 (PMC8795168; doi:10.1038/s41525-021-00278-6)
Supplement: Supplementary file 2 — Supplementary Information [file 41525_2021_278_MOESM2_ESM.pdf]

SUPPLEMENTARY MATERIAL

An evaluation of pipelines for DNA variant detection can guide a reanalysis protocol to increase the diagnostic ratio of genetic diseases. *npj Genomic Medicine.* Raquel Romero[1,2], Lorena de la Fuente[1,3], Marta Del Pozo-Valero[1,2], Rosa Riveiro-Álvarez[1,2], María José Trujillo-Tiebas[1,2], Inmaculada Martín-Mérida[1,2], Almudena Ávila-Fernández[1,2], Ionut-Florin Iancu[1,2], Irene Perea-Romero[1,2], Gonzalo Núñez-Moreno[1,3], Alejandra Damián[1,2], Cristina Rodilla[1], Berta Almoguera[1,2], Marta Cortón[1,2], Carmen Ayuso[1,2,*], Pablo Mínguez[1,2,3,*]

[1]Department of Genetics, Health Research Institute-Fundación Jiménez Díaz University Hospital, Universidad Autónoma de Madrid (IIS-FJD, UAM), Madrid, Spain.

2. Center for Biomedical Network Research on Rare Diseases (CIBERER), Instituto de Salud Carlos III, Madrid, Spain

3. Bioinformatics Unit, Health Research Institute-Fundación Jiménez Díaz University Hospital, Universidad Autónoma de Madrid (IIS-FJD, UAM), Madrid, Spain.

[*]Carmen Ayuso (cayuso@fjd.es) and Pablo Minguez (pablo.minguez@quironsalud.es) are corresponding authors.

SUPPLEMENTARY FIGURES

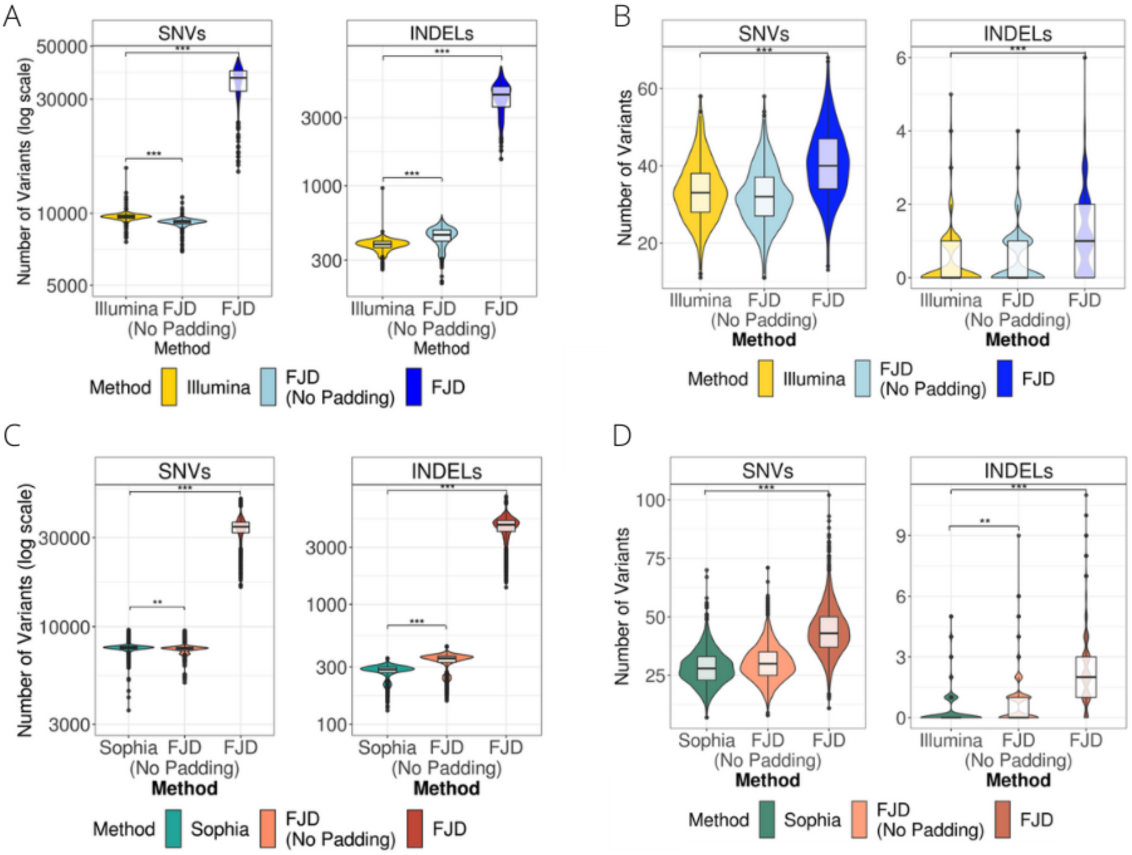

Supplementary Figure 1. Distribution of the number of variants (SNVs and INDELs) detected by commercial pipelines (Illumina and Sophia) and the FJD-pipeline in the samples from the general cohort. **(A)** Distribution of the number of variants (SNVs and INDELs) detected by Illumina and FJD pipelines. **(B)** Distribution of the number of clinically relevant variants (SNVs and INDELs) detected by Illumina and FJD pipelines. **(C)** Distribution of the number of variants (SNVs and INDELs) detected by Sophia and FJD pipelines. **(D)** Distribution of the number of clinically relevant variants (SNVs and INDELs) detected by Sophia and FJD pipelines. Clinically relevant variants are selected as having: GnomAdg_AF_POPMAX<0.05 and CADD Phred Score<15. The distributions were compared by applying a t-test. Significant levels are (*) p-value<0.05; (**) p-value<0.01; (***) p-value<0.001.

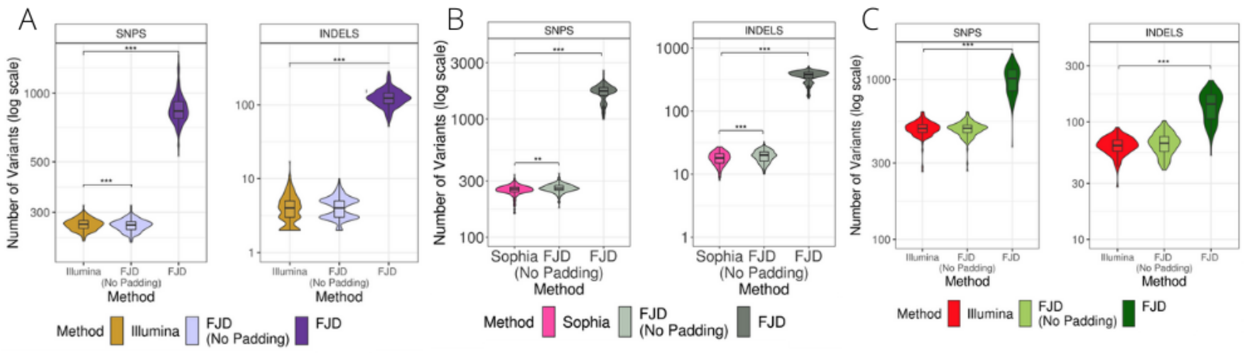

Supplementary Figure 2. (A) Distribution of the number of variants (SNVs and INDELs) detected by the Illumina and FJD pipelines in the cancer cohort sequenced using the TruSight Cancer panel (TSCa). **(B)** Distribution of the number of variants (SNVs and INDELs) detected by the Sophia-pipeline and the FJD-pipeline in the cancer subcohort sequenced using the Hereditary Cancer Solution panel (HCS). **(C)** Distribution of the number of variants (SNVs and INDELs) detected by Illumina and FJD pipelines in the cardiogenetics cohort sequenced using the Nextera Rapid Capture panel (NRC). The distributions were compared by applying a t-test. Significant levels are (*) p-value<0.05; (**) p-value<0.01; (***) p-value<0.001.

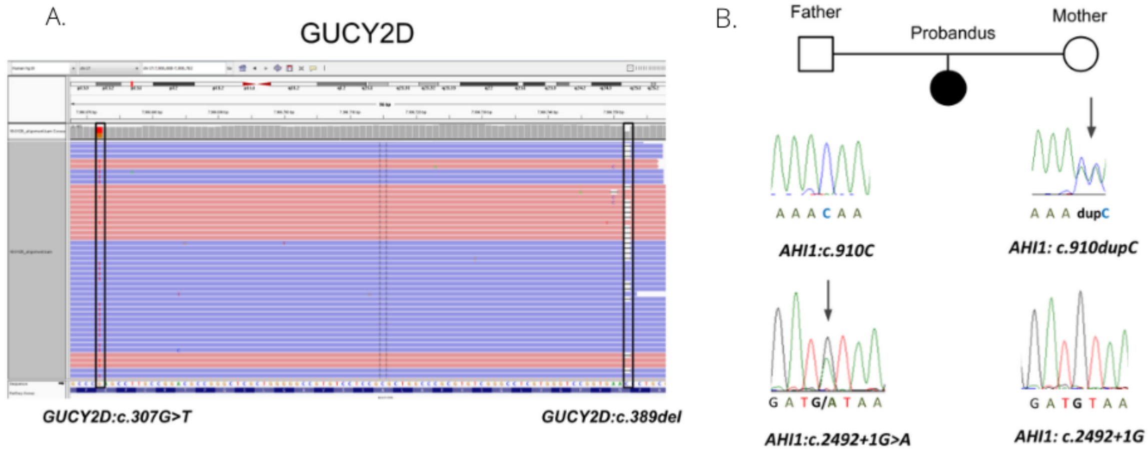

Supplementary Figure 3. Extra confirmatory evidence for two causal variants detected uniquely by the FJD-pipeline in cases solved by reassessment. **(A)** Variants found in gene GUCY2D, c.389del detected uniquely by the FJD-pipeline and c.307G>T detected by both Illumina and FJD pipelines. Mapping data show that both causal variants appear in trans. **(B)** Familiar segregation confirms the second variant in gene AHI1 (c.910dup) detected uniquely by the FJD-pipelines. Variant AHI1:c.910dup was found in the mother by sanger sequencing, whereas the other variant AHI1:c.2492+1G>A (detected in a previous analysis by the Illumina-pipeline) had been found in the father, both asymptomatic.

SUPPLEMENTARY TABLES

Supplementary Table 1. Number of variants detected by: the commercial pipeline (Illumina or Sophia), the FJD-pipeline, or both, for the totality of samples classified by the type of sequencing used: TruSightOne (TSO), Clinical Exome Solution (CES), Nextera Rapid Capture (NRC), TruSight Cancer (TSCa), and the Hereditary Cancer Solution by Sophia Genetics (HCS).

NUMBER OF VARIANTS						
	FJD-NoPadding vs Commercial			FJD-Default vs Commercial		
Panel	Both	FJD-NoPadding	Commercial	Both	FJD-Default	Commercial
TSO	4,686,073	216,571	419,094	4,686,077	16,975,017	419,090
CES	23,446,248	950,849	707,796	23,475,422	98,543,531	678,622
NRC	67,390	3,206	3,424	67,390	78,012	3,424
TSCa	78,861	1,326	4,019	78,861	188,130	4,019
HCS	64,451	6,468	2,365	64,474	504,809	2,342

Supplementary Table 2. Fold change of the mean of the number of variants (SNVs and INDELs) detected per sample by the FJD-pipeline (No-Padding and Default) and commercial pieplines. Samples from the general cohort sequenced by TruSightOne (TSO) and Clinical Exome Solution (CES) are considered.

MEAN FOLD CHANGE			
Sequencing techinque	Reanalysis by	SNVs	INDELs
TSO	FJD (No padding)	0.94	1.14
	FJD (Default)	3.73	10.97
CES	FJD (No padding)	0.98	1.21
	FJD (Default)	4.37	16.17

Supplementary Table 3. P-values obtained by the t-tests used in the comparisons of the distribution of SNVs and INDELs detected by Illumina-pipeline and FJD-pipeline, with (Default) and without (No padding) extended regions included. Samples sequenced from the general cohort sequenced with the TrueSightOne (TSO) panel are considered.

P-VALUES		
	**TSO**	
	SNVs	INDELs
FJD (No padding)	3.26e-10	8.43e-08
FJD (Default)	2.50e-63	1.82e-57

Supplementary Table 4. Fold change of the mean of the number of variants (SNVs and INDELs) detected by the FJD-pipeline (No-Padding and Default) and the Illumina-pipeline, divided by genomic types of genomic regions. TSO is the sequencing technique applied and stands for TruSightOne.

MEAN FOLD CHANGE								
**TSO**								
		SPLICING	EXONIC	UPSTREAM	DOWNSTREAM	INTRONIC	3'UTR	5'UTR
SNVs	FJD (No padding)	0.95	0.95	0.90	0.89	0.94	0.96	0.97
	FJD (Default)	0.99	0.97	3.60	3.07	35.71	4.46	5.57
INDELs	FJD (No padding)	1.11	1.18	1.18	1.09	1.24	1.10	1.23
	FJD (Default)	1.17	1.27	8.32	7.64	47.78	3.41	6.27

Supplementary Table 5: P-values obtained by the t-tests used for the comparisons of the distributions of SNVs and INDELs detected by the Illumina-pipeline and the FJD-pipeline (No-Padding and Default), divided by types of genomic regions. TSO is the sequencing technique applied and stands for TruSightOne.

P -VALUES								
**TSO**								
		SPLICING	EXONIC	UPSTREAM	DOWNSTREAM	INTRONIC	3UTR	5UTR
SNVs	FJD (No padding)	3.11e-05	1.37e-06	2.95e-11	3.41e-13	0.0606	0.0026	0.0236
	FJD (Default)	0.4290	3.65e-04	6.85e-72	4.78e-71	7.52e-76	2.77e-70	1.28e-87
INDELs	FJD (No padding)	1.03e-05	2.46e-14	3.76e-07	0.0126	0.0077	0.0014	2.17e-08
	FJD (Default)	1.97e-11	2.23e-25	2.25e-62	1.30e-60	3.69e-65	1.56e-53	8.85e-68

Supplementary Table 6. Fold change of the mean of the number of clinically relevant variants (SNVs and INDELs) detected by the FJD-pipeline (No-Padding and Default) and the Illumina-pipeline. Clinically relevant variants are defined by the ClinVar database as those annotated as '*pathogenic', 'likely pathogenic', 'uncertain significance'* or a combination of just those categories, VUS are filtered by allele frequency (GnomAdg_AF_POPMAX<0.1). TSO is the sequencing technique applied and stands for TruSightOne.

MEAN FOLD CHANGE		
**TSO (Clinically relevant)**		
	SNP	INDEL
FJD (no padding)	0.95	1.28
FJD (Default)	1.22	2.99

Supplementary Table 7. P-values obtained by the t-tests used for the comparisons of the distributions of clincally relevant SNVs and INDELs detected by the Illumina-pipeline and the FJD-pipeline (No-Padding and Default). TSO is the sequencing technique applied and stands for TruSightOne. Clinically relevant variants are defined by the ClinVar database as those annotated as '*pathogenic', 'likely pathogenic', 'uncertain significance'* or a combination of just those categories, VUS are filtered by allele frequency (GnomAdg_AF_POPMAX<0.1).

P-VALUES		
**TSO (Clinically relevant)**		
	SNP	INDEL
FJD (no padding)	0.097	0.110
FJD (Default)	2.14E-08	6.05E-09

Supplementary Table 8. Fold change of the mean of the number of clinically relevant variants (SNVs and INDELs) detected by the FJD-pipeline (No-Padding and Default) and the Illumina-pipeline, divided by types of genomic regions. Clinically relevant variants are defined by the ClinVar database as those annotated as '*pathogenic', 'likely pathogenic', 'uncertain significance'* or a combination of just those categories, VUS are filtered by allele frequency (GnomAdg_AF_POPMAX<0.1). TSO is the sequencing technique applied and stands for TruSightOne.

MEAN FOLD CHANGE								
**TSO (Clinically relevant)**								
		SPLICING	EXONIC	UPSTREAM	DOWNSTREAM	INTRONIC	3'UTR	5'UTR
SNVs	FJD (No padding)	0.91	0.96	0.98	0.91	1.00	0.90	1.12
	FJD (Default)	0.95	0.96	1.50	1.25	3.97	2.43	3.94
INDELs	FJD (No padding)	0.46	1.14	0.45	0.58	2.04	4.80	7.00
	FJD (Default)	0.93	1.14	1.45	1.58	3.97	7.60	155.00

Supplementary Table 9: P-values obtained by the t-tests used for the comparisons of the distributions of clincally relevant SNVs and INDELs detected by the Illumina-pipeline and the FJD-pipeline (No-Padding and Default), divided by types of genomic regions. TSO is the sequencing technique applied and stands for TruSightOne. Clinically relevant variants are defined by the ClinVar database as those annotated as '*pathogenic', 'likely pathogenic', 'uncertain significance'* or a combination of just those categories, VUS are filtered by allele frequency (GnomAdg_AF_POPMAX<0.1).

P -VALUES								
**TSO (Clinically relevant)**								
		SPLICING	EXONIC	UPSTREAM	DOWNSTREAM	INTRONIC	3UTR	5UTR
SNVs	FJD (No padding)	0.3772	0.2484	0.9289	0.2755	0.8634	0.3673	0.5710
	FJD (Default)	0.6348	0.2484	0.0001	0.0382	6.05E-21	6.50E-14	4.92E-16
INDELs	FJD (No padding)	0.5492	0.7756	0.4730	0.4097	0.0035	0.4097	0.1583
	FJD (Default)	0.4825	0.7756	1	0.7022	2.34E-08	0.2508	5.14E-08

Supplementary Table 10. P-values obtained by the t-tests used in the comparisons of the distribution of SNVs and INDELs detected by the Sophia-pipeline and FJD-pipeline with (Default) and without extended regions (No padding). Samples sequenced with the Clinical Exome Solution (CES) panel are considered.

	P-VALUES	
	**CES**	
	SNVs	INDELs
FJD (No padding)	1.71e-03	2.12e-19
FJD (Default)	2.37e-81	2.79e-70

Supplementary Table 11. Fold change of the mean of the number of variants (SNVs and INDELs) detected by the FJD-pipeline (No-Padding and Default) and the Sophia-pipeline, divided by types of genomic regions. CES is the sequencing technique applied and stands for Clinical Exome Solution.

		MEAN FOLD CHANGE						
		**CES**						
		SPLICING	EXONIC	UPSTREAM	DOWNSTREAM	INTRONIC	3UTR	5UTR
SNVs	FJD (No padding)	1.09	1.07	1.07	1.03	1.02	1.14	1.12
	FJD (Default)	2.13	1.08	5.08	3.94	78.23	11.05	7.48
INDELs	FJD (No padding)	2.55	1.07	1.30	1.24	1.04	1.66	1.09
	FJD (Default)	3.84	1.14	11.02	12.33	147.61	25.96	7.36

Supplementary Table 12: P-values obtained by the t-tests used for the comparisons of the distributions of SNVs and INDELs detected by the Sophia-pipeline and the FJD-pipeline (No-Padding and Default), divided by types of genomic regions. CES is the sequencing technique applied and stands for Clinical Exome Solution.

		P -VALUES						
		**CES**						
		SPLICING	EXONIC	UPSTREAM	DOWNSTREAM	INTRONIC	3UTR	5UTR
SNVs	FJD (No padding)	0.9850	0.0012	0.1927	2.23e-13	6.62e-03	0.1421	0.3838
	FJD (Default)	1.89e-148	0.1953	8.40e-86	5.66e-92	6.89e-81	1.23e-92	5.41e-113
INDELs	FJD (No padding)	3.84e-62	0.5446	6.11e-11	2.04e-07	0.2251	4.37e-21	0.9249
	FJD (Default)	5.01e-75	0.0009	9.98e-74	1.49e-74	2.02e-79	1.56e-70	1.42e-83

Supplementary Table 13. Fold change of the mean of the number of clinically relevant variants (SNVs and INDELs) detected by the FJD-pipeline (No-Padding and Default) and the Sophia-pipeline. Clinically relevant variants are defined using the ClinVar database as those annotated as '*pathogenic', 'likely pathogenic', 'uncertain significance'* or a combination of just those categories, VUS are filtered by allele frequency (GnomAdg_AF_POPMAX<0.1). CES is the sequencing technique applied and stands for Clinical Exome Solution.

MEAN FOLD CHANGE		
**CES (Clinically relevant)**		
	SNP	INDEL
FJD (no padding)	1.07	2.29
FJD (Default)	1.56	8.18

Supplementary Table 14. Fold change of the mean of the number of clinically relevant variants (SNVs and INDELs) detected by the FJD-pipeline (No-Padding and Default) and the Sophia-pipeline, divided by types of genomic regions. CES is the sequencing technique applied and stands for Clinical Exome Solution. Clinically relevant variants are defined using the ClinVar database as those annotated as '*pathogenic', 'likely pathogenic', 'uncertain significance'* or a combination of just those categories, VUS are filtered by allele frequency (GnomAdg_AF_POPMAX<0.1).

MEAN FOLD CHANGE								
**CES (Clinically relevant)**								
		SPLICING	EXONIC	UPSTREAM	DOWNSTREAM	INTRONIC	3'UTR	5'UTR
SNVs	FJD (No padding)	1.073	1.150	1.130	1.140	1.055	1.302	1.197
	FJD (Default)	1.818	1.153	2.089	1.886	13.650	8.913	7.810
INDELs	FJD (No padding)	5.770	1.761	0.705	1.050	1.000	1.333	58.4
	FJD (Default)	8.044	1.763	1.199	3.515	4487	135.333	168

Supplementary Table 15. P-values obtained by the t-tests used in the comparisons of the distribution of clinicaly relevant SNVs and INDELs detected by the Sophia-pipeline and FJD-pipeline with (Default) and without extended regions (No padding). Samples sequenced with the Clinical Exome Solution (CES) panel re included. Clinically relevant variants are defined using the ClinVar database as those annotated as '*pathogenic', 'likely pathogenic', 'uncertain significance'* or a combination of just those categories, VUS are filtered by allele frequency (GnomAdg_AF_POPMAX<0.1).

P-VALUES		
**CES (Clinically relevant)**		
	SNP	INDEL
FJD (no padding)	0.1267	0.0085
FJD (Default)	7.61E-26	2.20E-19

Supplementary Table 16: P-values obtained by the t-tests used for the comparisons of the distributions of clinicaly relevant SNVs and INDELs detected by the Sophia-pipeline and the FJD-pipeline (No-Padding and Default), divided by types of genomic regions. CES is the sequencing technique applied and stands for Clinical Exome Solution. Clinically relevant variants are defined using the ClinVar database as those annotated as '*pathogenic', 'likely pathogenic', 'uncertain significance'* or a combination of just those categories, VUS are filtered by allele frequency (GnomAdg_AF_POPMAX<0.1).

P -VALUES								
**CES (Clinically relevant)**								
		SPLICING	EXONIC	UPSTREAM	DOWNSTREAM	INTRONIC	3UTR	5UTR
SNVs	FJD (No padding)	0.6723	0.0007	0.4278	0.1114	0.8971	0.3161	0.1696
	FJD (Default)	1.19E-07	0.0006	8.68E-13	5.80E-09	7.29E-26	9.32E-35	1.44E-26
INDELs	FJD (No padding)	0.0006	0.1219	0.8073	0.4097	1	1	0.0030
	FJD (Default)	3.20E-05	0.1219	0.8168	0.3968	4.72E-22	0.0040	3.86E-07

Supplementary Table 17. Fold change of the mean of number of variants (SNVs and INDELs) detected by the FJD-pipeline (No-Padding and Default) and commercial pipelines applied in samples sequenced by: TruSight Cancer (TSCa), Nextera Rapid Capture (NRC) and Hereditary Cancer Solution by Sophia Genetics (HCS).

MEAN FOLD CHANGE			
		SNVs	INDELs
TSCa	FJD (No padding)	0.99	1.02
	FJD (Default)	3.22	30.70
NRC	FJD (No padding)	0.99	1.05
	FJD (Default)	1.98	2.16
HCS	FJD (No padding)	1.03	1.07
	FJD (Default)	6.68	19.99

Supplementary Table 18. P-values obtained by the t-tests used in the comparisons of the distribution of SNVs and INDELs detected in hereditary cancer samples sequenced with the TruSight Cancer (TSCa) panel using the Illumina-pipeline and FJD-pipeline with (Default) and without extended regions (No padding) included.

P-VALUES		
**TSCa**		
	SNVs	INDELs
FJD (No padding)	2.73e-10	0.0694
FJD (Default)	1.21e-65	7.32e-55

Supplementary Table 19: P-values obtained by the t-tests used in the comparisons of the distribution of SNVs and INDELs detected in hereditary cancer samples sequenced with the Hereditary Cancer Solution by Sophia Genetics (HCS) panel using the Sophia-pipeline and FJD-pipeline with (Default) and without extended regions (No padding) included.

P-VALUES		
**HCS**		
	SNP	INDEL
FJD (No padding)	0.0034	4.04E-26
FJD (Default)	7.25E-76	2.06E-70

Supplementary Table 20: P-values obtained by the t-tests used in the comparisons of the distribution of SNVs and INDELs detected in cardiovascular disease samples sequenced with the Nextera Rapid Solution panel using the Illumina-pipeline and FJD-pipeline with (Default) and without extended regions (No padding) included.

P-VALUES		
**NRC**		
	SNP	INDEL
FJD (No padding)	0.6592	0.1328
FJD (Default)	7.77E-43	5.90E-34

Supplementary Table 21. Fold change of the mean of number of variants (SNVs and INDELs) detected by the FJD-pipeline (No-Padding and Default) and the Illumina-pipeline in hereditary cancer samples sequenced using TruSight Cancer panel (TSCa), divided by types of genomic regions.

MEAN FOLD CHANGE								
**TSCa**								
		SPLICING	EXONIC	UPSTREAM	DOWNSTREAM	INTRONIC	3'UTR	5'UTR
SNVs	FJD (No padding)	1.12	1.01	0.94	0.94	0.98	0.99	0.99
	FJD (Default)	8.42	1.05	2.71	2.41	7.71	4.66	13.78
INDELs	FJD (No padding)	1.89	0.69	0.91	1.05	1	0.5	1
	FJD (Default)	8.80	0.77	7.09	9.09	1921.13	352.25	3.83

Supplementary Table 22:P-values obtained by the t-tests used in the comparisons of the distribution of SNVs and INDELs detected by the FJD-pipeline (No-Padding and Default) and the Illumina-pipeline in hereditary cancer samples sequenced using TruSight Cancer panel (TSCa), divided by types of genomic regions.

P -VALUES								
**TSCa**								
		SPLICING	EXONIC	UPSTREAM	DOWNSTREAM	INTRONIC	3UTR	5UTR
SNVs	FJD (No padding)	0.3287	0.2005	1.43e-09	9.05e-09	3.91e-02	0.8975	0.8857
	FJD (Default)	3.71e-90	5.17e-04	4.56e-57	3.35e-29	8.52e-67	9.79e-58	2.80e-60
INDELs	FJD (No padding)	8.00e-09	0.1268	0.1290	0.3302	0.3197	0.5631	1.000
	FJD (Default)	1.23e-45	0.4118	1.71e-28	6.35e-50	5.40e-56	6.06e-46	0.0034

Supplementary Table 23: Fold change of the mean of number of variants (SNVs and INDELs) detected by the FJD-pipeline (No-Padding and Default) and the Sophia-pipeline in hereditary cancer samples sequenced using Hereditary Cancer Solution panel (HCS), divided by types of genomic regions.

MEAN FOLD CHANGE								
**HCS**								
		SPLICING	EXONIC	UPSTREAM	DOWNSTREAM	INTRONIC	X3UTR	X5UTR
SNVs	FJD (No padding)	1.15	1.04	0.98	0.99	1	1.01	0.96
	FJD (Default)	3.22	1.05	4.91	4.88	289.28	101.88	8.44
INDELs	FJD (No padding)	3.16	0.43	0.97	0.53	41.33	1	1
	FJD (Default)	3.33	0.50	5.85	9.41	3063.46	619.5	1.47

Supplementary Table 24: P-values obtained by the t-tests used in the comparisons of the distribution of SNVs and INDELs detected by the FJD-pipeline (No-Padding and Default) and the Sophia-pipeline in hereditary cancer samples sequenced using Hereditary Cancer Solution panel (HCS), divided by types of genomic regions.

P-VALUES								
**HCS**								
		INTRONIC	EXONIC	DOWNSTREAM	SPLICING	3UTR	UPSTREAM	5UTR
SNVs	FJD (No padding)	0.9617	8.15E-04	0.4985	4.40E-07	0.9853	0.5672	0.5205
	FJD (Default)	1.66E-67	9.22E-06	4.34E-12	7.50E-59	2.12E-75	1.36E-54	9.49E-24
INDELs	FJD (No padding)	1.30E-12	7.75E-23	2.87E-27	6.026E-59	0.9988	0.0641	0.679
	FJD (Default)	1.04E-62	1.29E-18	1.32E-57	1.32E-57	1.02E-60	7.99E-27	7.09E-09

Supplementary Table 25: Fold change of the mean of number of variants (SNVs and INDELs) detected by the FJD-pipeline (No-Padding and Default) and the Illumina-pipeline in cardiogenetic samples sequenced using Nextera Rapid Capture panel (NRC), divided by types of genomic regions.

MEAN FOLD CHANGE								
		**NRC**						
		SPLICING	EXONIC	UPSTREAM	DOWNSTREAM	INTRONIC	3UTR	5UTR
SNVs	FJD (No padding)	1.01	0.99	1.02	0.96	0.99	0.99	0.98
	FJD (Default)	3.6	0.99	2.15	1.80	12.03	0.99	0.99
INDELs	FJD (No padding)	7	1.12	1.2	1.03	1	1.04	1.20
	FJD (Default)	570	1.12	3.17	1.76	9.28	1.04	1.24

Supplementary Table 26: P-values obtained by the t-tests used in the comparisons of the distribution of SNVs and INDELs detected by the FJD-pipeline (No-Padding and Default) and the Illumina-pipeline in cardio genetic samples sequenced using Nextera Rapid Capture panel (NRC), divided by types of genomic regions.

P-VALUES								
		**NRC**						
		INTRONIC	EXONIC	DOWNSTREAM	SPLICING	3UTR	UPSTREAM	5UTR
SNVs	FJD (No padding)	0.7228	0.7615	0.1129	0.9394	0.7868	0.4505	0.1752
	FJD (Default)	3.36E-52	0.7656	0.369E-31	2.36E-77	0.9094	7.55E-43	0.4633
INDELs	FJD (No padding)	0.9714	0.4850	0.5239	0.0311	0.1319	0.0011	0.0086
	FJD (Default)	9.64E-44	0.4850	1.52E-18	2.13E-32	0.1265	1.91E-25	0.0145

Supplementary Table 27: Summary of the variants previously found in the monoallelic arRD cases solved by reassessment.

Sample	Gene	Transcript	Nucleotide	Protein	Type	Inheritance	Zygosity	Phenotype	Region	ACMG	ACMG Criteria	**GnomAD AF**
18-0126	*GUCY2D*	NM_000180.3	c.307G>T	p.Glu103fsTer	SNV	AR	HET	Leber´s congenital Amaurosis	Exonic	Pathogenic	PVS1, PP3, PM2	-
16-0951	*CEP290*	NM_025114.4	c.4041G>A	p.Trp1347*	SNV	AR	HET	Leber´s congenital Amaurosis	Exonic	Pathogenic	PVS1, PP3, PM2	-
21-0476	*AHI1*	NM_017651.4	c.2429+1G>A	p.?	SNV	AR	HET	Joubert Syndorme	Exonic	Pathogenic	PVS1, PM2, PP3, PP5	9.89E-06
